# Supplementary figures and images for: Targeted genomic enrichment and sequencing of CyHV-3 from carp tissues confirms low nucleotide diversity and mixed genotype infections
Source: PeerJ. 2016 Sep 27;4:e2516. doi: 10.7717/peerj.2516 (PMC5045873; doi:10.7717/peerj.2516)

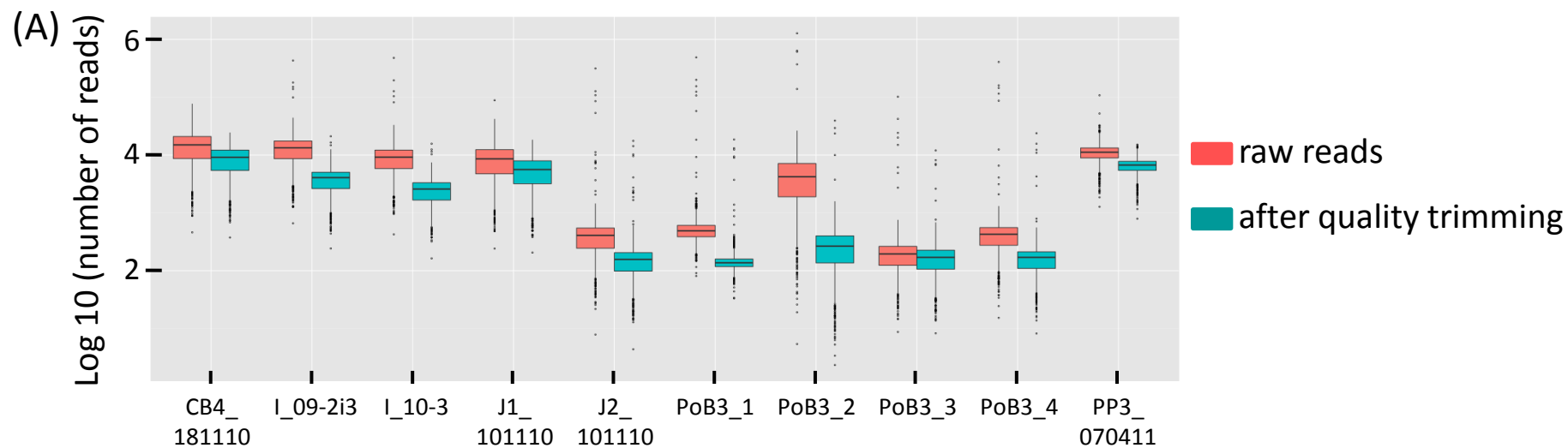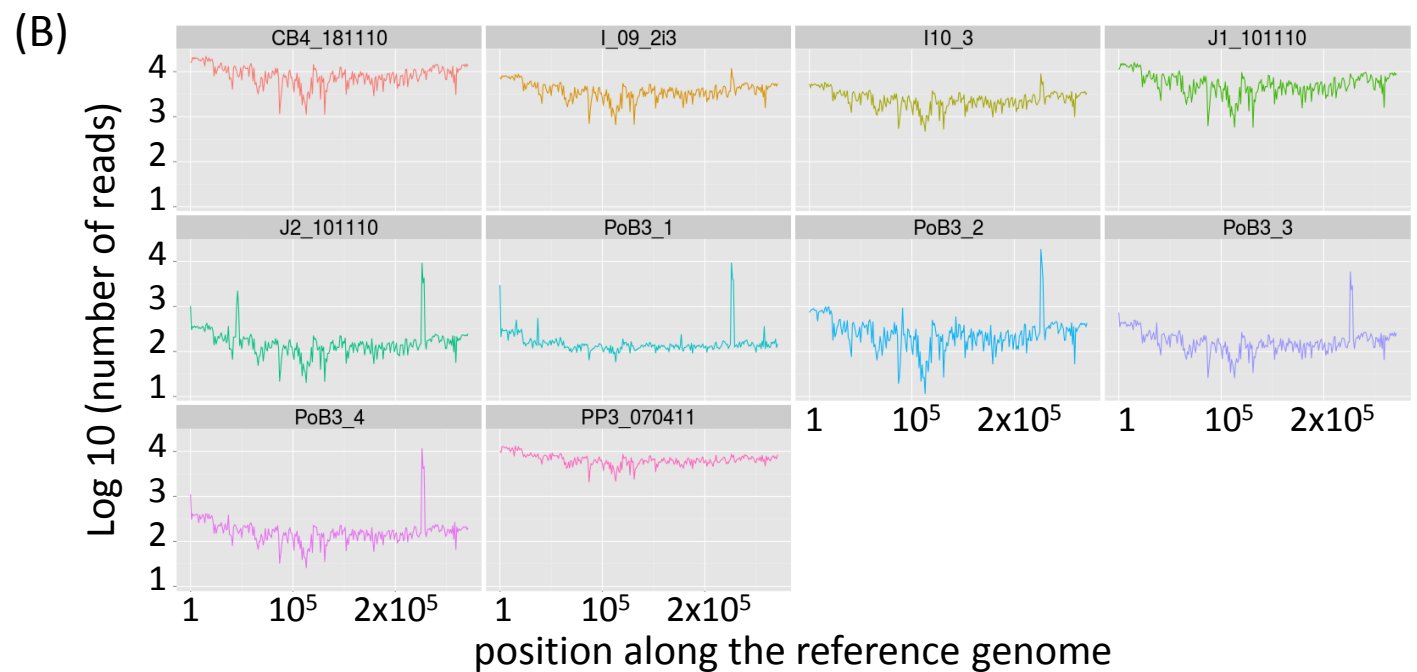

Supplement: Figure S1 — (A) Boxplot representing the depth distribution in log10 of raw reads (red) and filtered reads (blue), i.e. after Q30 mapping quality trimming and duplicate removal. (B) Per base sequencing depth along the KHV-J reference genome. The figure was created with the R package ggplot2. [file peerj-04-2516-s001.pdf]

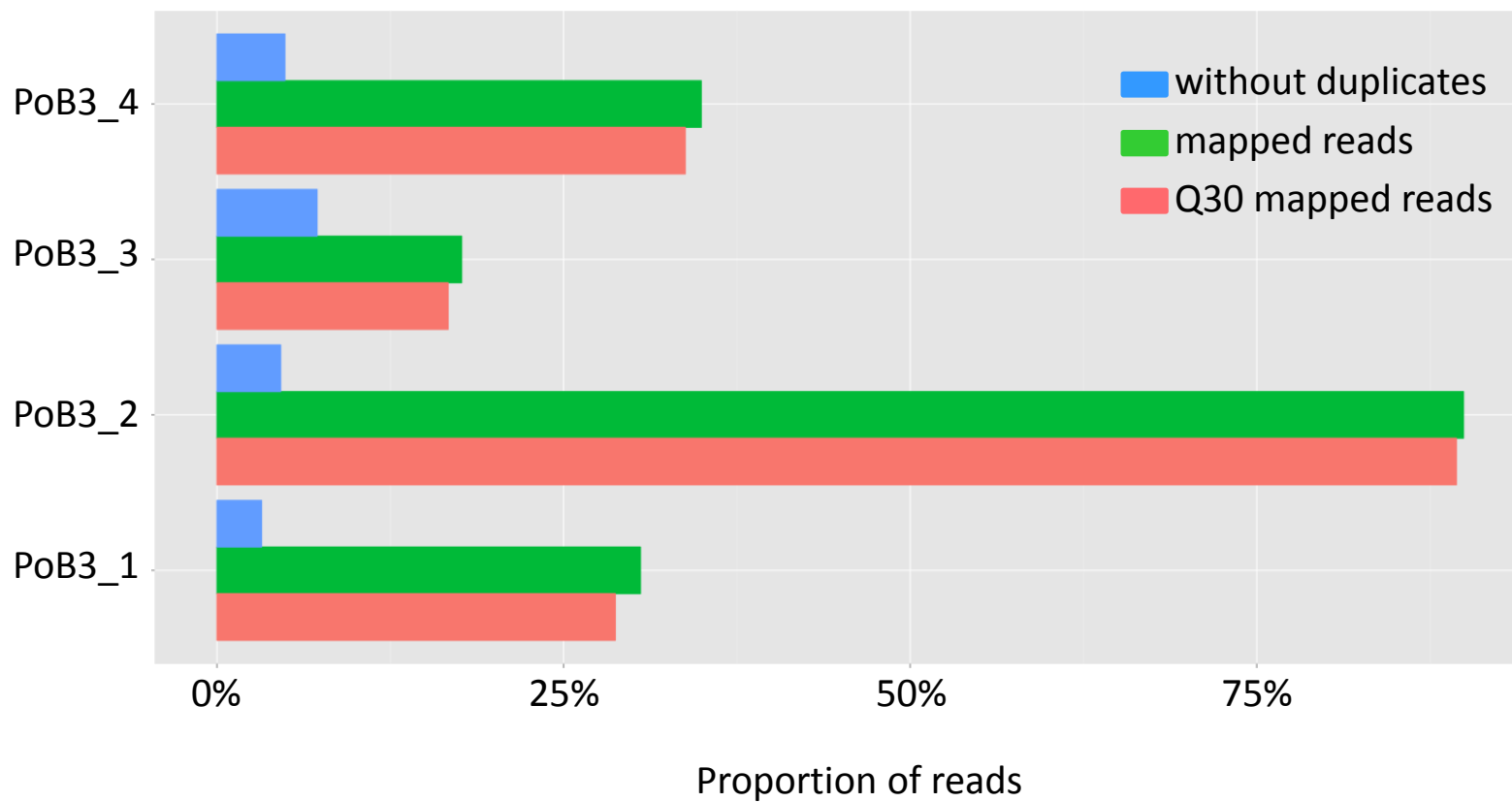

Supplement: Figure S2 — Proportion of mapped reads for PoB3 samples, expressed as the percent of reads that match a KHV sequence. Green bars: before quality trimming; red bars: after Q30 mapping quality trimming; blue bars: after duplicate removal. [file peerj-04-2516-s002.pdf]

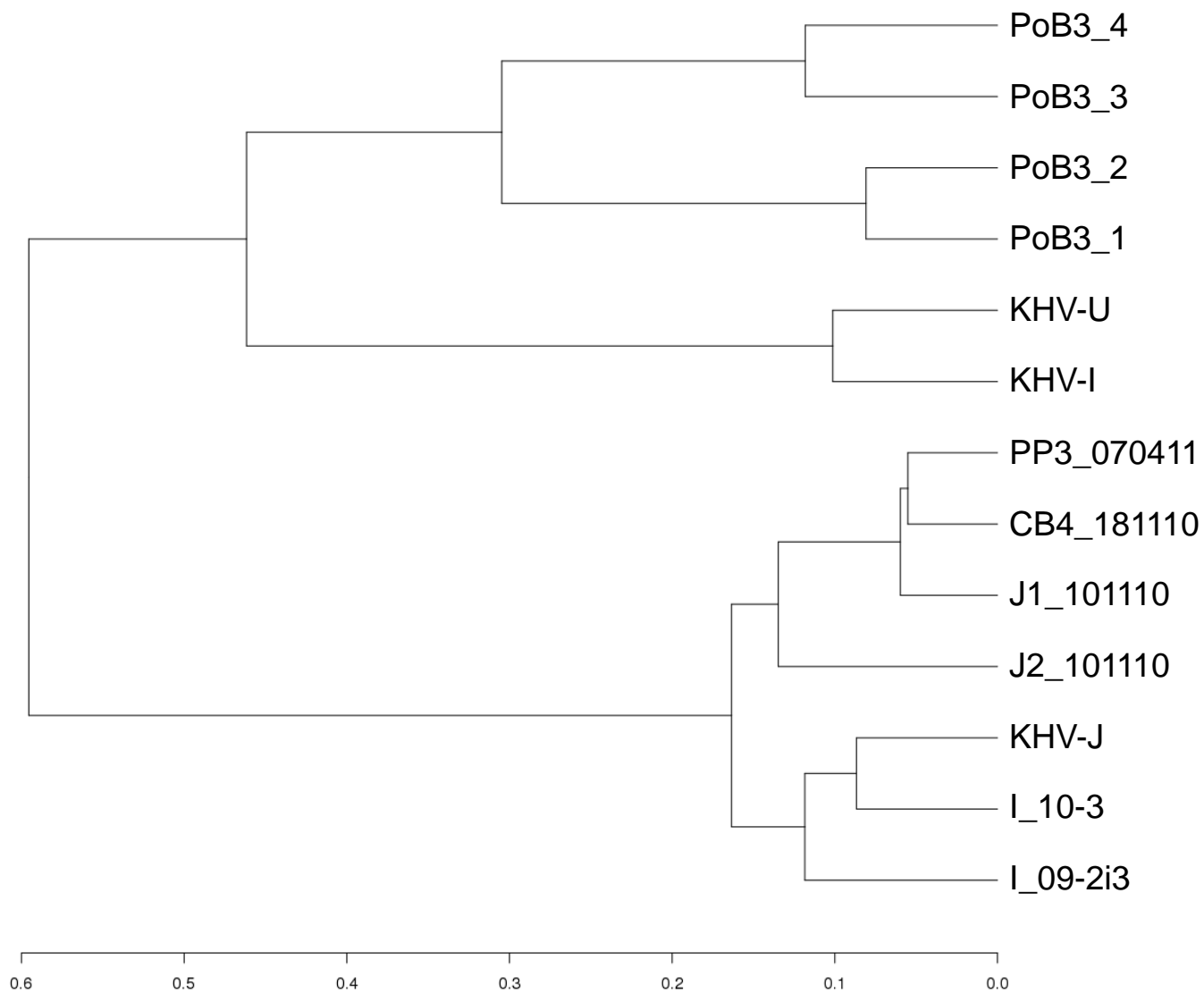

Supplement: Figure S3 — A distance matrix between samples was first computed using an Identity By State (IBS) distance. Specimens were then grouped into clusters using the R package hclust and the corresponding dendrogram plotted. The scale represents the maximumIBS-distance between two elements of linked clusters (branches in the tree). [file peerj-04-2516-s003.pdf]

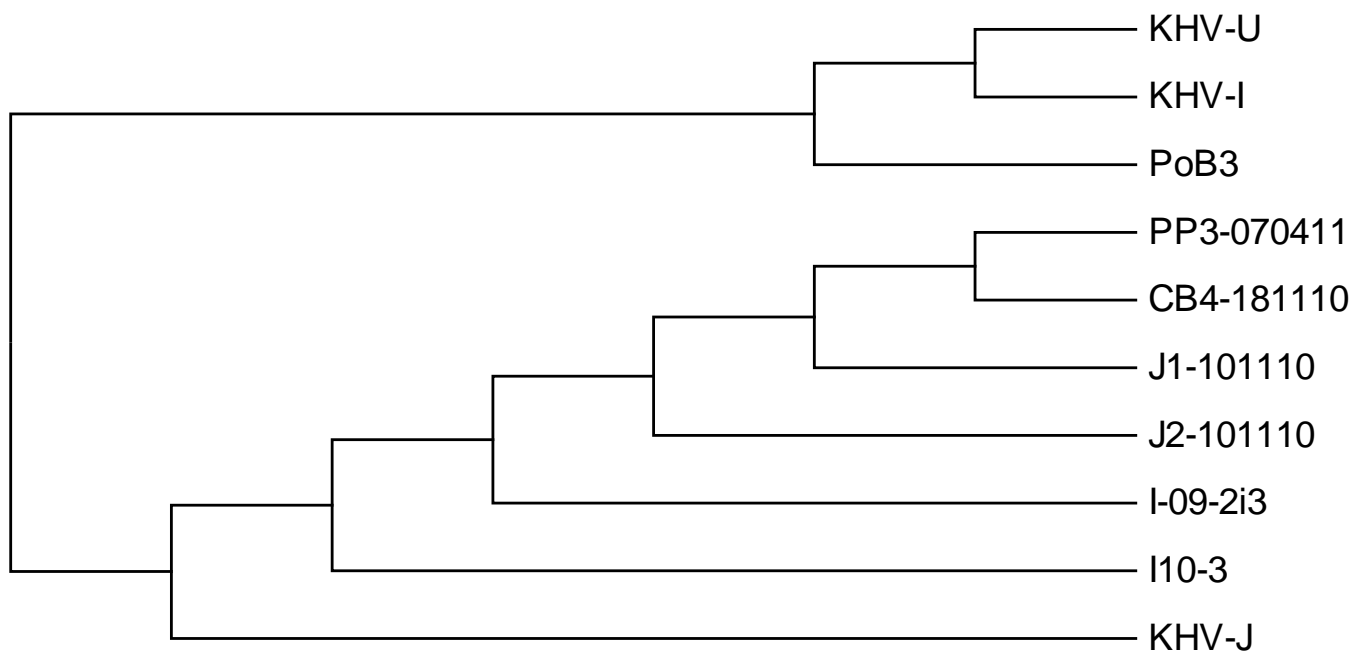

Supplement: Figure S4 — The tree was drawn to scale, and the branch lengths represent the number of base substitutions per site. [file peerj-04-2516-s004.pdf]
